# Safety assessment of intravenous immunoglobulins in pediatric population: A systematic review of adverse events

**DOI:** 10.1097/MD.0000000000048173

**Published:** 2026-04-17

**Authors:** Muteb Altowairqi, Naglaa M. Kamal, Abdulrahman Aljaber, Abdullah M. Alelyani, Mazen A. Alzaedi, Saad S.S. Aljuaid, Abdulelah S. Algethami, Mohammed A.M. Oshi, Ahmed S.A. Soliman

**Affiliations:** a Pediatric Department, AlHada Armed Forces Hospital, Taif, Kingdom of Saudi Arabia; b Pediatric Department, National Guard Hospital, Riyadh, Kingdom of Saudi Arabia; c Pediatric Department, Kasr Alainy Faculty of Medicine, Cairo University, Cairo, Egypt; d Gaafar Ibnauf Specialized Hospital, Khartoum, Sudan; e Pediatric Department, Faculty of Medicine, Benha University, Banha, Egypt.

**Keywords:** Adverse drug events, Intravenous Immunoglobulin, IVIG, Pediatrics

## Abstract

**Background:**

Intravenous immunoglobulin (IVIG) is a therapeutic intervention utilized in various disorders due to its antimicrobial, antiinflammatory, and immunomodulatory properties. The market offers a range of IVIG products, leading to variations in efficacy and safety profiles. This study aimed to systematically evaluate the safety profile of IVIG administration in pediatric patients.

**Methods:**

A systematic search was conducted on PubMed, Embase, and Cochrane databases using predetermined MeSH terms, covering the period from January 1, 2000, to October 15, 2022. Two independent reviewers performed relevance screening. Inclusion criteria encompassed English-language studies involving patients below 18 years of age who received IVIG treatment. Included studies were limited to peer-reviewed full-text publications with a minimum of 5 patients. A total of 8 studies met the inclusion criteria, encompassing 1088 pediatric patients.

**Results:**

Eight studies met the inclusion criteria (total 1088 pediatric patients). Five studies reported patient-level adverse event data suitable for pooling (688 patients). Among these 688 patients, 267 (38.8%) experienced adverse events (AEs). Among reported AEs, the most frequent were headache 68/267 (25.5%) and fever 47/267 (17.6%). Less common AEs included nausea 12/267 (4.5%) and fatigue 12/267 (4.5%). Four studies recorded a total of 2960 IVIG infusions, with 12.6% of infusions accompanied by AEs. The predominant AEs during infusions were headache (54.3%) and fever (29.4%). Cough (0.8%) and nausea (1.6%) were the least commonly observed AEs. Other AEs such as rash, vomiting, and abdominal pain were also reported. Of the 23 full-text articles reviewed, 8 were included. The remaining 15 were excluded due to the following reasons: 6 for duplicate data, 4 for inappropriate study design, 3 for not meeting the minimum number of patients, and 2 for incomplete adverse event reporting. These reasons are now detailed in the updated Preferred Reporting Items for Systematic Reviews and Meta-Analyses flow diagram.

**Conclusion:**

IVIG therapy demonstrates good tolerability in pediatric patients, with an overall favorable safety profile, aside from mild AEs associated with its administration. Serious AEs were infrequent and mainly observed in high-risk patients. Headache was the most prevalent AE, which seldom required hospitalization and could be effectively managed with acetaminophen. Other AEs were generally mild systemic reactions. Notably, AEs appear to be more frequent during the initial exposure to IVIG.

## 1. Introduction

Intravenous immunoglobulins (IVIGs) have gained significant recognition as a valuable treatment option for immune thrombocytopenia, Kawasaki disease, and primary immunodeficiencies. Manufactured from pooled human plasma, IVIGs primarily consist of unmodified immunoglobulin G (IgG), with minimal amounts of immunoglobulin A (IgA) or immunoglobulin M (IgM).^[1]^ Apart from its role in combating infections through replacement therapy, immunoglobulin has demonstrated antiinflammatory and immunomodulatory effects, making it a versatile therapeutic intervention through its antiinflammatory and immunomodulatory mechanisms.^[2]^ IVIG usage has expanded rapidly across different medical fields, including neurology, hematology, rheumatology, and dermatology, with proven efficacy in various conditions.^[2]^

IVIG can be administered at a “replacement dose” of 400 to 600 mg/kg/month for antibody deficiencies and at a higher dose (2 g/kg) as an “immunomodulatory” agent for immune and inflammatory disorders.^[3]^ Since its introduction in 1952, immunoglobulin replacement has been the standard therapy for primary immune deficiency diseases. Currently, there are over 25 approved IVIG preparations worldwide regulated by different authorities.^[3]^ These preparations exhibit differences in immunoglobulin and IgG subclass distribution, antibody content, approved maximum infusion rate, and side effects. Ideally, an optimal IVIG preparation would contain structurally and functionally intact immunoglobulin molecules, with a normal biological half-life and a balanced proportion of IgG subclasses.^[4]^

IVIG finds utility in the management of several immune-mediated and inflammatory diseases, such as Kawasaki syndrome and chronic inflammatory demyelinating polyneuropathy.^[1]^ It is also employed as a platelet-enhancing agent for the treatment of immune thrombocytopenia (ITP). The US Food and Drug Administration has approved the use of IVIG for various conditions, including the treatment of primary immunodeficiencies, prevention of infections in patients with hypogammaglobulinemia and B-cell chronic lymphocytic leukemia, prevention of coronary artery aneurysms in Kawasaki disease, prevention of infections and acute graft versus host disease after bone marrow transplantation, reduction of serious bacterial infection in children with human immunodeficiency virus, and increase of platelet count in idiopathic thrombocytopenic purpura.^[5,6]^

Despite the evident benefits of IVIG therapy, concerns persist regarding the occurrence of adverse events (AEs). Most IVIG reactions are mild and encompass symptoms such as backache, abdominal pain, nausea, chills, rhinitis, low-grade fever, myalgia, and headaches. However, more serious AEs, including anaphylaxis, renal, cardiovascular, central nervous system, and hematological events, have been reported during or shortly after IVIG infusion.^[7]^

## 2. Methods

A systematic review of the literature was conducted following the Preferred Reporting Items for Systematic Reviews and Meta-Analyses checklist. The review process involved 2 independent reviewers, ensuring consensus and minimizing discrepancies.

### 2.1. Data sources and search strategy

A comprehensive search was performed in 3 electronic databases: PubMed, Embase, and the Cochrane Library. The search encompassed studies published from January 1, 2000, to October 15, 2022. MeSH terms were utilized to optimize search accuracy. In PubMed, the search strategy included the following terms: (“immunoglobulins, intravenous” [MeSH Terms]) OR (“immunoglobulins” [All Fields] AND “intravenous” [All Fields]) OR (“intravenous immunoglobulins” [All Fields] OR “ivig” [All Fields]) AND (“adverse effects” [MeSH Subheading]) OR (“adverse” [All Fields] AND “effects” [All Fields]) OR (“adverse effects” [All Fields]) OR (“side” [All Fields] AND “effects” [All Fields]) OR (“side effects” [All Fields]) AND (“pediatrics” [All Fields] OR “pediatrics” [MeSH Terms]) OR (“pediatrics” [All Fields] OR “pediatric” [All Fields] OR “pediatric” [All Fields]). Similar search strategies were employed in Embase and the Cochrane Library. In addition to the electronic search, a manual search was conducted to ensure comprehensive coverage. No direct contact was made with any authors. The inclusion criteria were limited to studies published in the English language.

### 2.2. Study selection criteria

A set of predefined criteria was employed to evaluate the eligibility of studies for inclusion in the review. The study population was limited to children ranging in age from 0 months to 18 years who received treatment with IVIG. Peer-reviewed full-text publications with a minimum of 5 patients were considered for analysis. The study design encompassed both retrospective and prospective studies, including observational, randomized, and nonrandomized designs. Duplicate data, case reports, and series comprising fewer than 5 patients were excluded from the analysis to ensure robustness and reliability of the findings.

### 2.3. Data extraction

Data abstraction was performed for the studies that fulfilled the inclusion criteria. The extracted information encompassed the year of publication, country of origin, study characteristics, number of subjects, and details regarding side effects, including immediate and delayed AEs. Patient co-morbidities were not consistently reported across studies and were therefore excluded to maintain uniformity in data extraction. To evaluate the risk of bias in the included studies, the Cochrane methods were employed as a standardized approach for assessing the quality and potential sources of bias.^[8]^ This rigorous evaluation ensured the robustness and reliability of the data obtained from the selected studies.

## 3. Results

**Figure 1.**
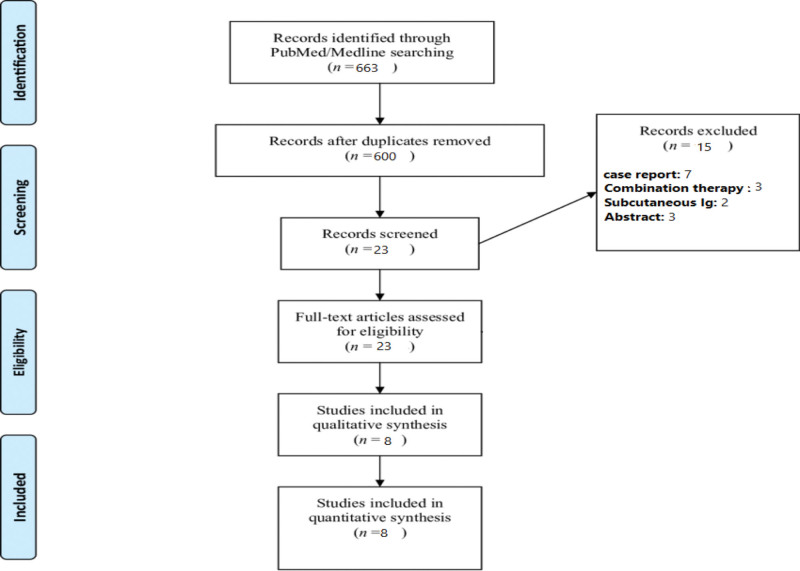
Flow diagram depicting the study inclusion process following the Preferred Reporting Items for Systematic Reviews and Meta-Analyses (PRISMA) guidelines.

A comprehensive literature search identified 663 records. After removal of duplicates, 600 records were screened by title and abstract. Twenty-three full-text articles were assessed for eligibility. Fifteen studies were excluded for the following reasons: duplicate data (n = 6), inappropriate study design (n = 4), fewer than 5 patients (n = 3), and incomplete adverse event reporting (n = 2). Ultimately, 8 studies met the inclusion criteria and were included in the qualitative synthesis (Fig. 1).

These selected publications encompassed pediatric patients who received IVIG, irrespective of whether it was their 1st exposure or not. The analysis focused on side effects that were consistently reported in at least 2 or more publications. The publication dates of the included studies ranged from 2012 to 2021, and the studies originated from various countries, including Japan, Egypt, Turkey, Iran, Canada, Qatar, and Australia (Table 1).^[9–16]^

**Table 1:** The study characteristics.

	Study	Year	Country	Patients number	Study design
1	Kubota	2020	Japan	104	Retrospective cohort study
2	Elalfy	2017	Egypt	48	Open-label study
3	Ibis	2020	Turkey	145	Retrospective cross-sectional study
4	Esmaeilzadeh	2021	Iran	363	Cohort study
5	Manlhiot	2008	Canada	135	Observational study
6	Elajez	2019	Qatar	120	Retrospective chart review study
7	Kaba	2017	Turkey	115	Cross sectional study
8	Singh-Grewal	2006	Australia	58	Prospective study
9	Ballow	2016	USA	24	Multicenter open labeled study
10	Melamed	2016	USA	25	Multicenter open labeled study
11	Ochs	2018	USA & Europe	36 divided into NGAM01 (25) and NGAM05 (11)	Multicenter open labeled study

### 3.1. AEs

AEs associated with IVIG administration were defined as symptoms occurring from the 1st day of IVIG infusion to 7 days after its completion. The majority of reported AEs were nonserious in nature. These included fever, headache, rash, vomiting, nausea, abdominal pain, cough, and fatigue. Among the reviewed studies, only a small number of serious events were documented, accounting for 2% of the patients observed in the study conducted by Elajez et al.^[14]^ The most frequently reported adverse reactions were fever and headache, followed by vomiting.

Where possible, we analyzed whether AEs were attributable to IVIG itself or the patients’ underlying conditions. For instance, Elajez et al.^[14]^ found renal insufficiency largely in at-risk patients, suggesting comorbidity influence. Additionally, studies such as Kubota et al^[16]^ showed a predominance of delayed AEs (79.5%).

### 3.2. Overall, 53% were immediate and 47% delayed

Out of **1088** patients from 8 included studies (Table 1),^[9–16]^ **5 studies** reported patient-level AE data suitable for pooled analysis (**688** patients). Among these 688 patients, **267 (38.8%**) experienced AEs (Table 2).^[9–11,13,16]^ Headache was the most common AE (**68/267; 25.5%**) followed by fever (**47/267; 17.6%**).

**Table 2:** Adverse events with IVIG infusion in relation to number of patients.

Reference	Age mean (range)	Indication for IVIG	Total adverse events	Fever	Headache	Rash	Vomiting	Nausea	Abdominal pain	Fatigue	Other adverse events number (example)
Kaba et al^[9]^	9 (2–16)	Idiopathic thrombocytopenic purpura	29 (25.2%)	13 (11.3%)	7 (6.1%)	2 (1.7%)	10 (8.7%)	2 (1.7%)	0	0	2 (wheezing, Atrial fibrillation)
Singh-Grewal et al^[10]^	10 (5–17)	Immunomodulation	26 (44.8%)	4 (6.7%)	15 (25.9%)	4 (6.7%)	0	4 (6.7%)	8 (13.8%)	12 (20%)	14 (fatigue, lethargy)
Elalfy et al^[11]^	7 (3–12)	Idiopathic thrombocytopenic purpura	16 (33.3%)	2 (4.2%)	8 (16.7%)	0	4 (8.3%)	0	0	0	2 (lethargy)
Esmaeilzadeh et al^[13]^	8 (1–17)	Inborn errors of immunity	157 (43.2%)	12 (3.3%)	23 (6.3%)	0	2 (0.5%)	0	0	0	75 (muscle pain, chills)
Kubota et al^[16]^	10 (6–14)	Neurological disorders	39 (37.5%)	16 (15,4%)	15 (14.4%)	13 (12.5%)	10 (9.6%)	6 (5.8%)	5 (4.8%)	0	13 (myalgia, chills)
Ballow^[17]^	11 (6–16)	Primary immunodeficiency	20 (83.3%)	7 (29.2%)	10 (41.7%)	0 (0%)	2 (8.3%)	2 (8.3%)	2 (8.3%)	0 (0%)	21 (headache, lethargy)
Church et al^[18]^	10 (4–16)	Primary immunodeficiency	14 (56%)	3 (12%)	11 (44%)	0 (0%)	0 (0%)	0 (0%)	3 (12%)	3 (12%)	25 (rigors, myalgia)
Ochs et al (NGAM01)^[19]^	10 (4–16)	Primary immunodeficiency	24 (96%)	5 (20%)	3 (12%)	0 (0%)	0 (0%)	0 (0%)	2 (8%)	2 (8%)	30 (nausea, chills)
Ochs et al (NGAM05)^[19]^	10 (4–16)	Primary immunodeficiency	10 (90.9%)	0 (0%)	1 (9.1%)	0 (0%)	0 (0%)	0 (0%)	1 (9.1%)	0 (0%)	6 (nausea, chills)
Total (773)			335 (43.3%)	62 (18.5%)	93 (27.7%)	19 (5.6%)	28 (8.4%)	14 (4.2%)	21 (6.3%)	17 (5.1%)	188

IVIG = intravenous immunoglobulin.

Nausea and fatigue were the least common AEs, occurring at rates of 4.5% each (Table 2).^[9–11,13,16]^ No severe AEs were recorded, except for 1 case of ventricular fibrillation in a patient with septic shock, as reported in the study by Kaba et al.^[9]^ Another case involved a patient experiencing chest pain and bronchospasm, who received corticosteroids and bronchodilators, as described in the study by Singh-Grewal et al.^[10]^ Additional studies included in the analysis but not previously discussed in the text were conducted by Ballow,^[17]^ Church et al,^[18]^ and Ochs et al (NGAM01 and NGAM05).^[19]^ These studies, while limited in size or scope, contributed relevant data on AEs in pediatric IVIG therapy.

Three additional studies^[17–19]^ were included in the qualitative synthesis but were not incorporated into the pooled patient-level adverse event analysis due to differences in reporting methodology. These studies primarily reported infusion-level adverse event rates or did not provide detailed patient-level symptom breakdowns suitable for aggregation.

Ballow^[17]^ reported safety outcomes in a pediatric cohort receiving 10% IVIG, with AEs predominantly mild and infusion-related. Church et al^[18]^ similarly demonstrated favorable tolerability, with most reactions classified as mild to moderate. Ochs et al^[19]^ evaluated IVIG 10% in pediatric primary immunodeficiency and reported a low incidence of serious AEs, with the majority of reactions occurring during early infusions.

Due to heterogeneity in outcome reporting (infusion-based versus patient-based incidence), these studies were analyzed descriptively rather than included in pooled calculations.

Our cohort findings align with Kubota et al pediatric IVIG-AE study, which in 104 patients observed similar patterns of immediate (≈10%) and delayed (≈38.5%) AEs, supporting the robustness of our AE classification and safety analysis.^[16]^

Four studies collectively reported 2960 IVIG infusions, among which 12.6% were associated with AEs.^[10,12,14,15]^ The most frequently reported adverse event was headache, accounting for 54.3% of infusions with AEs, followed by fever at 29.4%. Cough and nausea were the least common AEs, occurring at rates of 0.8% and 1.6% respectively. Other reported AEs included rash, vomiting, and abdominal pain. No severe AEs were recorded, except for 7 cases of anaphylaxis and 6 cases of renal insufficiency in the study conducted by Elajez et al,^[14]^ with 5 of the renal insufficiency cases involving patients at risk for this condition (Table 3).^[10,12,14,15]^

**Table 3:** Adverse events with IVIG infusion in relation to the number of infusions.

Reference	Age mean (range)	Primary indication	Total adverse events	Fever	Headache	Rash	Vomiting	Nausea	Abdominal pain	Cough	Fatigue	Anaphylaxis	Other adverse events
Singh-Grewal et al^[10]^	10 (5–17)	Immunomodulation	112 (32.5%)	4 (1.2%)	47 (13.6%)	9 (2.6%)	0	4 (1.2%)	8 (2.3%)	0	18 (5.2%)	0	Fatigue, abdominal pain
Ibis et al^[12]^	6 (2–15)	Primary immunodeficiency	129 (10.6%)	69 (5.7%)	96 (7.9%)	12 (1%)	31 (2.5%)	2 (0.16%)	0	3 (0.24%)	5 (0.41%)	0	Chills, hypotension
Elajez et al^[14]^	8 (1–17)	Immunodeficiencies	41 (11.8%	20 (5.8%)	7 (2%)	0	9 (2.6%)			0	0	7 (2%)	Nausea, renal injury
Manlhiot et al^[15]^	10 (5–16)	Juvenile dermatomyositis	92 (8.7%)	17 (1.6%)	53 (5%)	0	18 (1.7%)	0	0		5 (0.47%)	0	Lethargy
			374 (12.6%)	110 (29.4%)	203 (54.3%)	21 (6%)	58 (15.5%)	6 (1.6%)	8 (2.1%)	3 (0.8%)	28 (7.5%)	7 (1.9%)	

IVIG = intravenous immunoglobulin.

In summary, AE data have been differentiated based on whether they occurred per patient (38.8%) or per infusion (12.6%). This distinction helps clarify the true incidence of AEs per exposure unit.

## 4. Discussion

This systematic review examined the incidence and characteristics of AEs associated with IVIG administration in children based on published reports. The findings suggest that while AEs may occur frequently, the majority of them are mild and manageable. Several studies included in this review shed light on the incidence and types of AEs observed in different patient populations.

Kubota et al conducted a study on patients with neurological diseases and reported that 37.5% of the patients experienced AEs. Notably, delayed AEs were more prevalent (79.5%, 31/39) compared to immediate AEs (30.8%, 12/39).^[16]^ Singh-Grewal et al also reported a higher occurrence of delayed AEs.^[10]^

Among the AEs reported, fever and headache were the most common, while abdominal pain was less frequently observed.^[10]^

Elalfy et al focused on patients with ITP and categorized them into 3 groups. For this review, the results of groups A and B were considered. In these groups, 16 adverse drug-related events were reported, accounting for 33.3% of the patients, with an equal number of events in each group. Headache (16.7%), vomiting (8.3%), and pyrexia (4.2%) were the most commonly reported adverse drug reactions, and the rates were comparable between the 2 groups. One patient in Group B experienced a severe headache, leading to an extended hospital stay. No unexpected AEs were documented.^[11]^

Ibis et al^[12]^ and Manlhio et al^[15]^ reported an incidence of AEs of 14.2% when considering the number of infusions and a rate of 44.8% when considering the number of patients. These findings suggest that the incidence of AEs is higher during the 1st infusion.^[12,13]^ Previous studies have also identified 1st-time IVIG therapy as a risk factor for AEs.^[20–23]^

Ibis et al^[12]^ evaluated 145 children with primary immunodeficiency receiving IVIG replacement, with a total of 1214 infusions administered. Among these patients, adverse reactions occurred in 65 (44.8%). Of the patients receiving their 1st IVIG infusion, adverse reactions were observed in 23 infusions (24.7%). Recurrent infusion adverse reactions were noted in 149 out of 1121 infusions (13.2%). Immediate adverse reactions accounted for 53% of the total adverse reactions, while delayed reactions accounted for the remaining 47%. The most frequently observed immediate adverse reactions were fever (3.9%) and headache (2.7%), whereas the most common delayed adverse reactions were headache (5.1%), vomiting (1.8%), and fever (1.7%). Overall, headache was the most common adverse event (7.8%), with 2 cases classified as severe.^[12]^

Esmaeilzadeh et al enrolled patients with human inborn errors of immunity and analyzed a total of 22,667 infusions administered to 363 patients. Among all patients, 43.3% experienced adverse reactions, and 5.9% of the infusions were associated with at least 1 adverse reaction. Muscle pain was the most frequent adverse event (15.5%), followed by chills (15.2%), headache (14.0%), and fever (13.5%), all of which were mild. The study also found that the incidence of AEs varied depending on the order of infusions, with a higher occurrence of adverse reactions observed during the initial infusions.^[13]^

The initial administration of IVIG has been associated with a higher incidence of adverse reactions compared to subsequent infusions. The Immune Deficiency Foundation conducted a study indicating that 34% of adverse reactions to IVIG occur during the 1st infusion. However, after 2 or 3 infusions with the same product, additional adverse reactions become less frequent. Therefore, it is recommended to administer the 1st IVIG infusion slowly, using a 3% or 5% solution, starting at a rate of 0.5 to 1.0 mg/kg/min.^[5]^

A study by Manlhiot et al, focused on patients with juvenile dermatomyositis and reported that out of 38 patients, 25 (66%) experienced adverse reactions in 92 (9%) IVIG infusions.^[15]^ None of these reactions were life-threatening. Adverse events were more commonly observed with the initial infusion compared to subsequent ones (16% vs 9%).^[15]^ The increased frequency of AEs during the initial infusion may be attributed to subsequent premedication in patients intolerant to the 1st infusion.^[15]^ The most frequently reported reactions included fever, headaches, nausea or vomiting, and lethargy.^[15]^ Overall, the study concluded that IVIG therapy is generally well-tolerated and a safe option for children with juvenile dermatomyositis.^[15]^

Elajez et al conducted a study involving 345 pediatric patients who received IVIG at Hamad General Hospital in 2014.^[14]^ The study found that IVIG had a good safety profile, with 88.1% of infusions (304/345) completed without any documented AEs.^[14]^ Among the AEs recorded, fever was the most commonly documented (5.8%), followed by chills (2.6%), vomiting (2.6%), and headache (2%).^[14]^ Notably, 7 patients experienced hypersensitivity reactions despite being premedicated with paracetamol and diphenhydramine and receiving IVIG according to the recommended infusion protocol.^[14]^ Other AEs included hypotension, chills, nausea or vomiting, and renal injury.^[14]^

Kaba et al conducted a Turkish study involving 115 patients with various conditions, including primary immunodeficiencies, ITP, Kawasaki disease, secondary immunosuppression, and passive immunization. Immediate AEs were observed in 25.2% of the patients receiving IVIG.^[9]^ The most common immediate AEs reported were fever (11.8%), vomiting (8.7%), and headache (6.1%).^[9]^ Other AEs included rash, nausea, wheezing, and atrial fibrillation.^[9]^ Most of the reactions were mild and could be managed by reducing the infusion rate or using medications such as antihistamines, paracetamol, and low-dose corticosteroids.^[9]^

In a study by Singh-Grewal et al, AEs following IVIG therapy were investigated in patients with immunodeficiency and immunomodulation.^[10]^ Out of 58 subjects, 26 (44.8%) reported at least 1 adverse event. Immediate reactions were observed in 10.3% of the children, while delayed reactions occurred in 41.4%. Twelve infusions (3.5%) were associated with immediate AEs, and 72 infusions (20.9%) were linked to delayed AEs.^[10]^ The most common delayed adverse event was headache, reported by 24.1% of patients and 12.8% of infusions. Fatigue and abdominal pain were also frequently reported.^[10]^

It is important to note that Ibis et al^[12]^and Manlhio et al^[15]^ calculated the percentage of AEs based on the number of infusions rather than the number of patients. The calculation of adverse event percentages in those 2 studies focused on the number of infusions rather than the number of patients. The frequency of AEs ranged from 25.2%^[9]^ to 44.8%^[10]^ across the included patient-level studies.

These results emphasize the significance of adverse event monitoring during IVIG infusions and highlight the need for strategies to minimize these occurrences.

## 5. Conclusion

In conclusion, the administration of IVIG in pediatric patients generally proves to be a safe and well-tolerated therapeutic approach. However, it is important to note that although the majority of AEs associated with IVIG are mild and manageable, there is still a possibility of rare serious reactions. Hence, diligent monitoring during the infusion process is imperative. Among the reported AEs, headache was the most frequently observed, but it rarely necessitated hospitalization and could be effectively managed using acetaminophen. Additionally, other AEs primarily consisted of mild systemic reactions. Importantly, it appears that the incidence of AEs is higher during the initial exposure to IVIG. These findings underscore the importance of closely monitoring patients during their 1st IVIG administration and implementing appropriate measures to minimize any potential risks.

## Author contributions

**Conceptualization:** Muteb Altowairqi.

**Formal analysis:** Muteb Altowairqi, Abdullah M. Alelyani, Mazen A. Alzaedi, Saad S.S. Aljuaid, Abdulelah S. Algethami.

**Investigation:** Naglaa M. Kamal, Mohammed A.M. Oshi.

**Methodology:** Ahmed S.A. Soliman.

**Writing – original draft:** Muteb Altowairqi, Naglaa M. Kamal, Abdulrahman Aljaber, Saad S.S. Aljuaid, Abdulelah S. Algethami, Mohammed A.M. Oshi, Ahmed S.A. Soliman.

**Writing – review & editing:** Muteb Altowairqi, Naglaa M. Kamal, Ahmed S.A. Soliman.
